# Biomarkers of COVID-19 severity may not serve patients with polycystic ovary syndrome

**DOI:** 10.1186/s12967-021-02723-7

**Published:** 2021-02-11

**Authors:** Abu Saleh Md Moin, Manjula Nandakumar, Thozhukat Sathyapalan, Stephen L. Atkin, Alexandra E. Butler

**Affiliations:** 1 Diabetes Research Center (DRC), Qatar Biomedical Research Institute (QBRI), Hamad Bin Khalifa University (HBKU), Qatar Foundation (QF), PO Box 34110, Doha, Qatar; 2 Academic Endocrinology, Diabetes and Metabolism, Hull York Medical School, Hull, UK; 3 Royal College of Surgeons in Ireland Bahrain, Adliya, Kingdom of Bahrain

**Keywords:** Biomarkers, Polycystic ovary syndrome, COVID-19, SARS-CoV-2, Platelet degranulation, Coagulation factors

**To the Editor:**

In a cohort of patients with differing severity of COVID-19 disease, including non-survivors, plasma proteomic analysis identified biomarkers of COVID-19 disease progression [1]. The top pathways identified by Shu et al. were those of platelet degranulation and the complement and coagulation cascades [1]. These identified pathways were complementary to another recent study comparing COVID-19 disease and control subjects, where proteomic panels also identified biological pathways involved in platelet degranulation and the coagulation cascade [2]. Whilst the comparison with absolute disease-free normality is relevant, an increasing proportion of the population have insulin resistant states with associated metabolic conditions; an example of such a metabolic condition is polycystic ovary syndrome (PCOS) where it has been shown that protein expression patterns may differ compared to those without PCOS [3]. Notably, in PCOS, platelet aggregation enhancement together with aberrant diminished plasma fibrinolytic activity potentially giving rise to enhanced thrombosis has been described [4, 5], with markers of coagulation being enhanced [6].

For a protein biomarker to be of value, there needs to be a clear discrimination between normal and disease condition levels. Therefore, platelet degranulation and the complement and coagulation cascade proteomic analysis was performed in women with and without PCOS to compare with these pathways described in COVID-19 disease [1].

243 subjects (146 PCOS and 97 control women) were recruited to the local PCOS biobank (ISRCTN70196169) [3] in the Department of Endocrinology, Hull and East Yorkshire Hospitals NHS Trust. The Rotterdam consensus diagnostic criteria were used to diagnose PCOS. Proteins that were described for platelet degranulation (18 of 27 proteins) and the complement and coagulation cascades (16 of 19 proteins) [1] were measured using the Slow Off-rate Modified Aptamer (SOMA)-scan plasma protein measurement [7], shown in Table 1. Statistics were performed using Graphpad Prism 8.0.

**Table 1:**
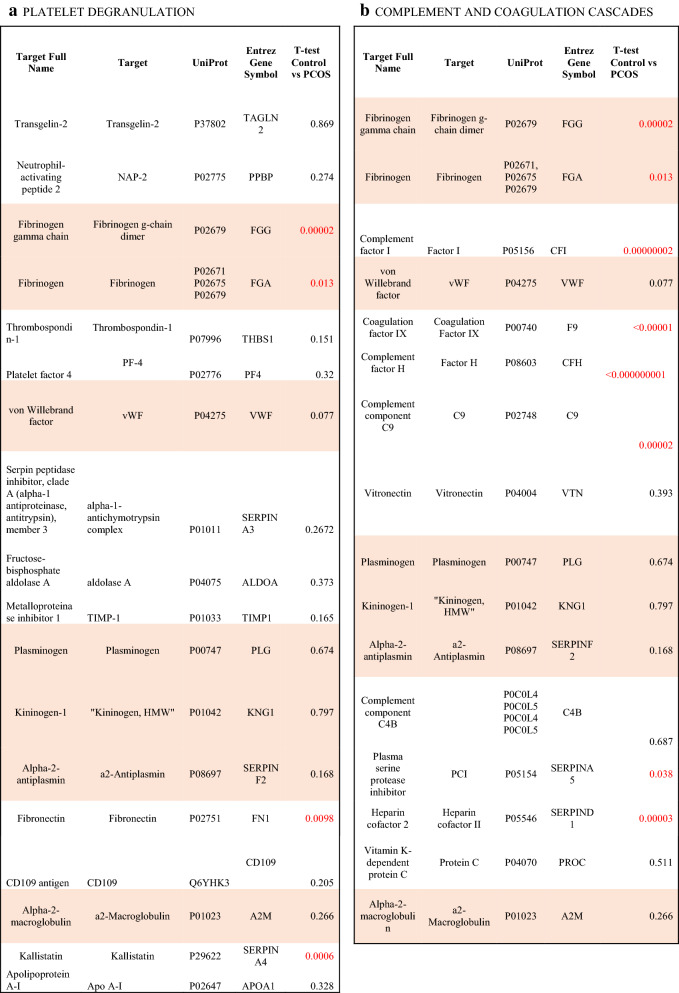
Proteins identified as being altered in COVID-19 disease categorized according to A, platelet degranulation; B, complement and coagulation cascades in non-COVID infected polycystic ovary syndrome (PCOS) and control women

Students’ t-test was used to determine differences between protein levels. Proteins that differed significantly (p < 0.05) are shown in red font. Proteins that are common to both the platelet degranulation and the complement/coagulation cascades are shaded in orange

As reported previously [3], whilst cohorts were age-matched, the PCOS women differed in having increased systolic and diastolic blood pressure and waist circumference (p < 0.05), together with increased insulin resistance, increased androgens and C-reactive protein (CRP) (p < 0.001), indicative of metabolic dysfunction.

For the 46 protein biomarkers described by Shu et al. [1], 34 were available for measurement in the Somalogic platform: 4 of 18 were found to differ in PCOS for platelet degranulation [fibrinogen-gamma chain (p = 0.00002), fibrinogen (p = 0.013), fibronectin (p = 0.0098) and kallistatin (p = 0.0006)], whilst 8 of 16 proteins for complement and coagulation cascade (fibrinogen-gamma chain (p = 0.00002), fibrinogen (p = 0.013), complement factor 1 (p = 0.00000002), coagulation factor IX (p < 0.00001), complement factor H (p < 0.000000001), complement component C9 (p = 0.00002), plasma serine protease inhibitor (p = 0.038) and heparin cofactor 2 (p = 0.00003) (Table 1). Moreover, those proteins that significantly differed between PCOS and controls share a close relationship to one another, as shown by the protein–protein interaction tool STRING (Search Tool for the Retrieval of Interacting Genes) pathways (Fig. 1).

**Fig. 1:**
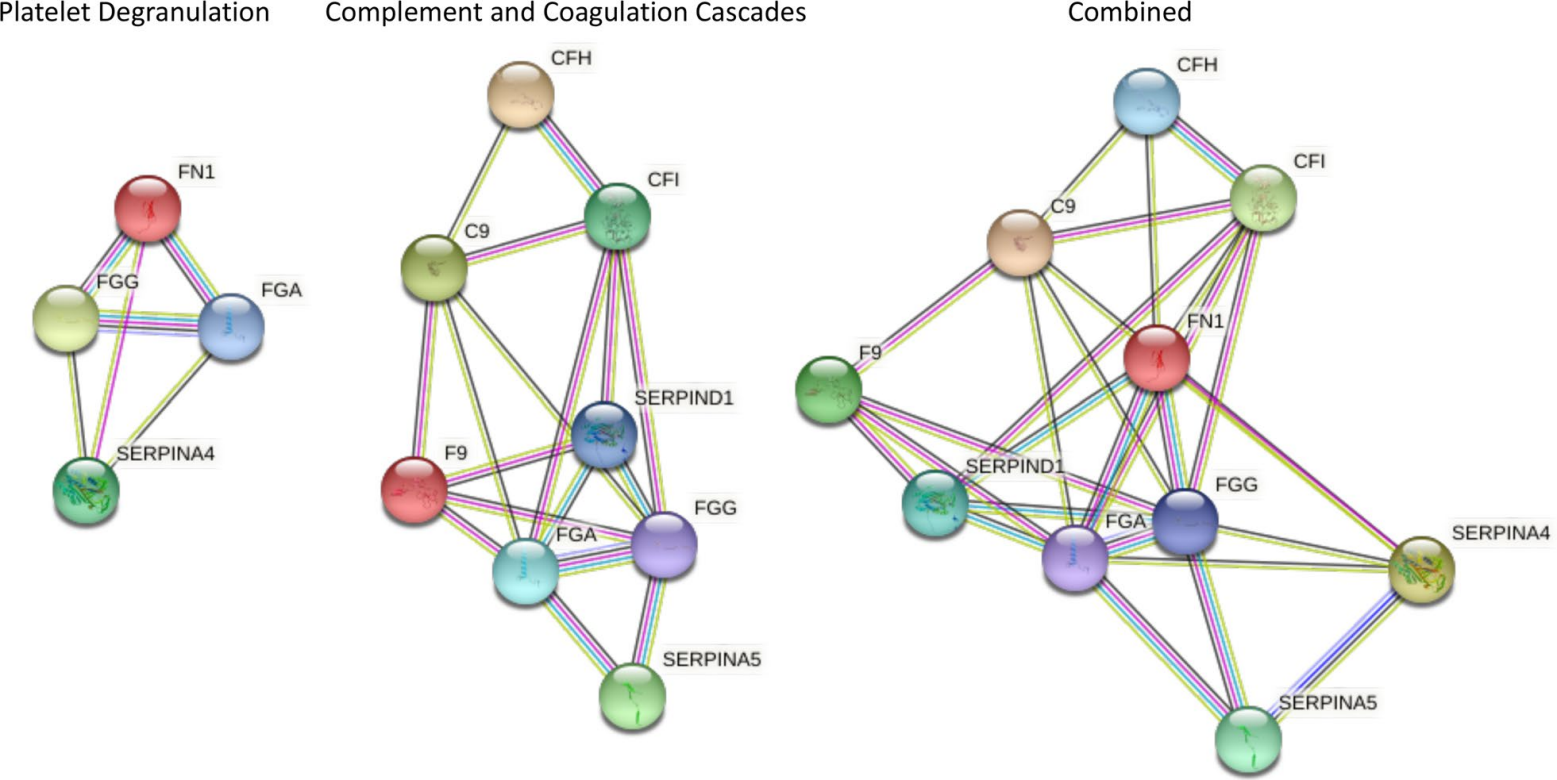
The protein–protein interaction tool STRING 11.0 (Search Tool for the Retrieval of Interacting Genes) was used to visualize the significantly different proteins in PCOS compared to controls, and for all of the proteomic proteins in COVID-19 disease severity described by others [1] (https://string-db.org/). Interactions between proteins are evidence-based and collated from databases, experiments, neighborhood, gene fusion, co-occurrence, text mining, co-expression, and homology. Here, we determined the relationships between the platelet degranulation (**a**) and complement and coagulation cascade proteins (**b**) presented in the study by Shu et al. [1] that were significantly different between non-COVID infected PCOS and control women. **a** Platelet degranulation proteins that differed significantly between PCOS and control women, indicating their relationship to one another. **b** Complement and coagulation cascade proteins that differed significantly between PCOS and control women, indicating their relationship to one another. **c** Combined platelet degranulation and complement and coagulation cascade proteins that differed significantly in PCOS, indicating their relationships to one another

The significant difference seen in PCOS compared to controls indicates the need for validation of such markers in the non-COVID-19 infected population before they can be considered as biomarkers for COVID-19 and its severity. Notably, based on the indication that COVID-19 severity can be related to these markers, their detection in a PCOS COVID-19 positive patient may give a false impression of severity, potentially leading to the introduction of inappropriate therapy; conversely, the detection of these specific markers in women with PCOS may actually indicate that more proactive intervention is required, as these women may have a propensity for increased COVID-19 disease severity [8].

Limitations of the study include that the Somalogic panel did not include all of the proteins that were previously reported, and the proteomic analysis differed so may not be directly comparable to the Shu et al. study [1] or others [2]. Nonetheless, the majority of proteins were common to both proteomic platforms.

In conclusion, 12 of 34 protein biomarkers contained within the platelet degranulation and complement and coagulation cascades and purported to indicate disease progression in patients infected with COVID-19, differed between non-COVID-19 infected PCOS and control women. This indicates that validation of such proposed COVID-19 specific biomarkers is a necessity, although it is unclear if this places PCOS women at increased risk of more severe COVID-19 disease.

## Data Availability

All the data for this study will be made available upon reasonable request to the corresponding author.
